# A Case Report of Chronic Epipharyngitis With Chronic Fatigue Treated With Epipharyngeal Abrasive Therapy (EAT)

**DOI:** 10.7759/cureus.54742

**Published:** 2024-02-23

**Authors:** Ito Hirobumi

**Affiliations:** 1 Otolaryngology, Ito ENT Clinic, Funabashi, JPN

**Keywords:** autonomic balancing action, heart rate variability analysis, orthostatic test, autonomic function tests, salivary α-amylase activity, salivary cortisol, endocrine function tests, chronic epipharyngitis, eat, me/cfs

## Abstract

A case of myalgic encephalomyelitis/chronic fatigue syndrome (ME/CFS) with chronic epipharyngitis was treated with epipharyngeal abrasive therapy (EAT). The symptoms of ME/CFS improved along with the improvement of chronic epipharyngitis. The patient was followed up with endocrine and autonomic function tests. Endocrine function tests included salivary cortisol and salivary α-amylase activity. Salivary α-amylase activity was stimulated by EAT. EAT improved the diurnal variability of salivary cortisol secretion. Autonomic function tests included heart rate variability analysis by orthostatic stress test. EAT normalized parasympathetic and sympathetic reflexes over time and regulated autonomic balance. Based on the improvement of symptoms and test results, EAT was considered effective for ME/CFS. A literature review was conducted on the mechanism of the therapeutic effect of EAT on ME/CFS.

## Introduction

Epipharyngeal abrasive therapy (EAT) is a treatment for chronic epipharyngitis. EAT consists of nasally and orally abrading the diseased epipharyngeal mucosa with a cotton swab soaked in a 1% zinc chloride solution [1]. In this case, a patient with chronic epipharyngitis who complained of chronic fatigue was treated with EAT, and the chronic fatigue improved. This patient had been diagnosed with ME/CFS at a clinic specializing in ME/CFS in Tokyo, but the presence of chronic epipharyngitis was not clear at first. The diagnosis of chronic epipharyngitis was confirmed by endoscopy at our clinic, and ME/CFS improved after treatment with EAT, suggesting that chronic epipharyngitis, which was latent in the patient, may have been the cause of the chronic fatigue.

In this case, endocrine and autonomic function tests were performed to evaluate the therapeutic effect of EAT on chronic fatigue and elucidate the mechanism of EAT's effect. The endocrine function tests included salivary cortisol measurement [2] and salivary α-amylase activity (SAA) measurement [3-4]. Autonomic function tests were performed by observing changes in blood pressure and heart rate during the orthostatic stress test. Heart rate variability analysis was also performed to evaluate changes in parasympathetic and sympathetic nervous activity [5]. The results showed that the patients were sensitive to external stimuli and that EAT improved their endocrine and autonomic functions. The literature discusses the mechanism of the effect of EAT on chronic fatigue in relation to ME/CFS.

## Case presentation

A 42-year-old woman with chronic fatigue of unknown origin, which began to interfere with her daily life and employment about six months ago, visited an outpatient clinic specializing in ME/CFS in Tokyo in February 2015, with chronic fatigue as the main complaint. At the time of the visit, she faced difficulty going out and spent more than 50% of the day lying in bed at home. She was diagnosed with ME/CFS according to the diagnostic criteria for chronic fatigue syndrome by the Japan Agency for Medical Research and Development (AMED) research group (formerly the Ministry of Health, Labor, and Welfare research group) [6]. She underwent treatment involving high-concentration intravenous vitamin C, dehydroepiandrosterone (DHEA), and nutritional therapy with oral magnesium supplements. However, there was no improvement in her symptoms.

The patient had a history of perennial allergic rhinitis caused by house dust and mites and had a habit of sniffing the nose since childhood. The patient had been aware of an abnormal sensation near the epipharynx but had not received any specific treatment because of the absence of a sore throat or posterior rhinorrhea. There was no family history of note. The primary physician referred the patient to our clinic in September 2015 based on reports that EAT is useful as a treatment for ME/CFS [1,7-8]. At the time of the initial visit to our clinic, the patient complained of tinnitus and insomnia as well as chronic fatigue.

**Figure 1:**
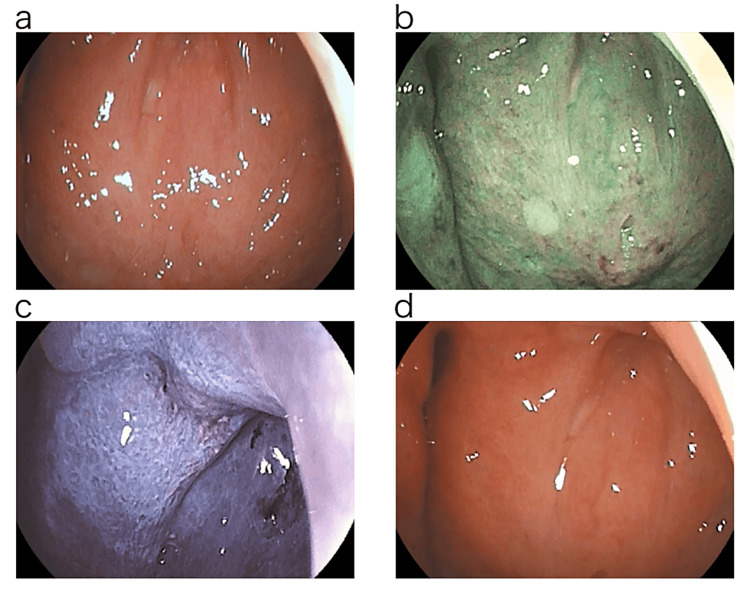
Endoscopic findings. (a) Endoscopic findings with normal light. The entire epipharyngeal mucosa was erythematous and swollen. The orifice of the Eustachian tube was obstructed, and pressure on the Rosenmuller fossa was observed. No crusts were observed. (b) Endoscopic findings with bandlimited light (optical enhancement mode 1). Internal hemorrhage on the mucosal surface. Deep vascular dilatation and congestion of the mucosa, mucosal thickening, and abscesses at the Tornwalt site. (c) EAT findings: Immediate Whitening Phenomenon was observed on the epipharyngeal mucosa immediately after EAT. Hemorrhage from a portion of the epipharyngeal mucosa was observed. (d) Endoscopic findings with normal light after EAT treatment. Redness and swelling of the epipharyngeal mucosa disappeared, and bleeding during rubbing was no longer observed. EAT, epipharyngeal abrasive therapy

The patient underwent nasopharyngeal endoscopy to confirm the presence of chronic epipharyngitis in our clinic. The examination was performed using a band-limited light endoscopy system (Pentax EPK-i7000 video processor, VNL11-J10 video scope with a tip outer diameter of ø3.5 mm). Normal light observation revealed diffuse erythema and swelling of the epipharyngeal mucosa from the posterior wall of the nasopharynx to the canal and lateral walls. The orifice of the Eustachian tube was swollen and obstructed, and there was compression of the Rosenmuller fossa. No crust adhesions were observed (Figure 1a). Observation using Optical Enhancement Mode 1, with band-limited light, enhances the contrast between the mucosal surface and deep vessels, making them easier to distinguish. The reddish-brown or blackish-brown color on the mucosal surface in this case was considered to indicate internal hemorrhage. The green color of the deep mucosa was considered to indicate deep vascular dilatation or congestion [9]. Mucosal thickening and abscesses were observed at the Thornwald site (Figure 1b).

EAT is a therapeutic diagnostic method for chronic epipharyngitis [1]. EAT was performed by nasal abrasion treatment with a Lutze swab soaked in 1% zinc chloride solution followed by oral abrasion treatment with a Zermac pharyngeal wound cotton. Immediately after the zinc chloride solution was applied to the epipharyngeal mucosa, an Immediate Whitening Phenomenon [9] was observed on the epipharyngeal mucosa. At the time of EAT, bleeding was observed from a part of the epipharyngeal mucosa, and the patient was aware of severe pain. According to Tanaka's diagnostic criteria [9], the patient was diagnosed with chronic epipharyngitis with chronic fatigue in our clinic (Figure 1c).

EAT was started in September 2015, twice a week. Initially, fever in the 37 °C range and sore throat were observed the day after EAT. Home rest and recuperation were the basis of treatment, combined with nasal gargling with saline solution.

Since it has been reported that changes in the gut microbiome may be a cause of ME/CFS, drug therapy was not performed considering the effect on the gut environment [10]. With EAT, bleeding during epipharyngeal abrasion gradually decreased, and fever and sore throat gradually disappeared after EAT was administered. As chronic epipharyngitis improved, subjective symptoms such as tinnitus and insomnia improved, and chronic fatigue also improved. About three months after the start of treatment, the patient's fatigue disappeared. About four months after the start of treatment, the patient was able to lead a normal daily life. The patient was able to return to work in March 2016, about six months after the start of treatment. During the six-month treatment period before returning to work, EAT was performed a total of 54 times, which means that EAT was performed approximately twice a week.

**Figure 2:**
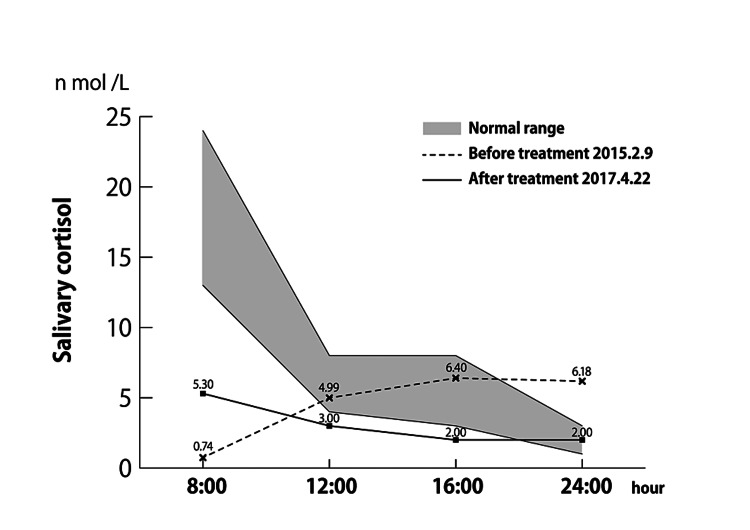
Diurnal variation in salivary cortisol. A graph comparing the diurnal variation values of salivary cortisol before and after EAT is shown. Before EAT, the morning surge disappeared and there was no diurnal fluctuation, while salivary cortisol increased in the afternoon. After EAT, the morning surge improved and the diurnal variation recovered. EAT, epipharyngeal abrasive therapy

The purpose of the study was to examine the effects of EAT on the hypothalamus-pituitary-adrenocortical system (HPA system) and the sympathetic-adrenal medulla system (SAM system). A salivary cortisol test was performed, and diurnal variations were recorded [2]. Diurnal variations of salivary cortisol were measured at a clinic specializing in ME/CFS in Tokyo in February 2015 before EAT was performed, and in April 2017 after the chronic epipharyngitis improved after EAT was performed. Figure 2 shows the diurnal variation of salivary cortisol measured at our clinic in April 2017 after the improvement of chronic epipharyngitis after EAT (measured by enzyme-linked immunosorbent assay (ELISA) by Bio Medical Laboratories [BML]). Comparing the diurnal variation of salivary cortisol before and after treatment, it was observed that the diurnal variation was improved by EAT.

Salivary cortisol is a useful indicator of chronic stress [2]. The diurnal variation disappeared before EAT was administered, and an increasing trend was observed in the afternoon. Elevated salivary cortisol in the afternoon can cause insomnia and other sleep disorders. It is possible that chronic epipharyngitis was a chronic stressor, causing abnormal salivary cortisol secretion, which may have contributed to the onset of ME/CFS. Before EAT treatment, cortisol secretion was abnormal, but as chronic epipharyngitis improved, the morning surge of salivary cortisol improved. Before treatment, cortisol secretion increased in the afternoon, but after treatment, the normal diurnal pattern of cortisol secretion was restored. Insomnia also improved with the improvement of chronic epipharyngitis, suggesting that EAT affects the HPA system and normalizes cortisol secretion.

**Figure 3:**
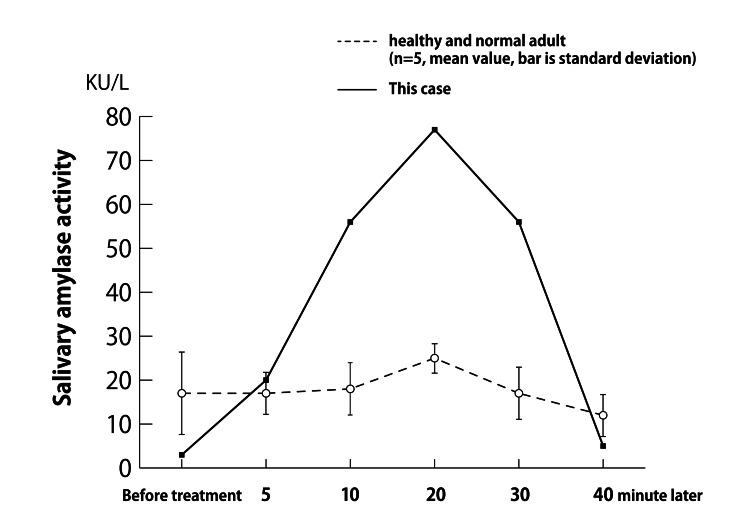
Change over time in salivary α-amylase activity. Changes in salivary α-amylase activity (SSA) over time after EAT administration are shown. A trend toward SAA prompting was observed compared to changes in healthy adults. EAT, epipharyngeal abrasive therapy

In April 2017, SAA was measured with a salivary amylase monitor (Nipro, Salivary Amylase Monitor Enzyme Analyzer). Changes over time before and after EAT were measured, and changes in the measured values compared to those of healthy adults (*n *= 5) are shown in Figure 3. SAA is used as a measure of acute stress and reflects sympathetic nerve function [3]. Cases showed a tendency for SAA to be stimulated by EAT stimulation compared to healthy adults. Cases were considered to have enhanced reactivity to acute stress compared to healthy controls. This was thought to indicate that the sympathetic nervous system is overactive when exposed to stress.

An active orthostatic stress test with electrocardiographic recordings was performed as an autonomic function test before EAT. The purpose of the autonomic function test was to confirm whether the case met the diagnostic criteria for orthostatic intolerance and determine its effect on the baroreceptor reflex (BR) [11]. Autonomic function tests were performed three times: in October 2015, immediately after starting EAT; in May 2016, after symptoms had improved due to EAT implementation and the patient had returned to work; and in April 2017, approximately one year after returning to work. Heart rate (HR) variability analysis software was Kiritsu Meijin by Crosswell.

For the standing test, the patient was first held in a resting sitting position for two minutes and then stood up. After standing, the standing position was held for two minutes. Next, the subjects were seated and held in a sitting position for one minute. The resting sitting phase was designated as Phase 1, the standing phase as phase 2, the standing phase as phase 3, and the seated phase as phase 4. The mean values of HR, systolic blood pressure (SBP), mean arterial pressure (MAP), and diastolic blood pressure (DBP) in each phase were The mean values were obtained as the endpoints.

Orthostatic dysregulation (OD) is the presence of an excessive drop in blood pressure that occurs when the patient assumes a standing position. OD is diagnosed when there is a drop in SBP of 20 mmHg or more, a drop in DBP of 10 mmHg or more, or both. Postural orthostatic tachycardia syndrome (POTS) is diagnosed when the heart rate increases to more than 120 beats per minute or by 30 beats per minute when the patient is moved from the supine to the upright position [11].

**Figure 4:**
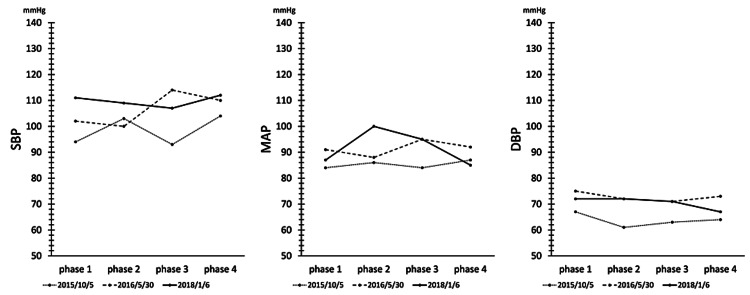
Changes in blood pressure during the active standing test. The patient was first held in a resting sitting position for two minutes before standing up. After standing, the standing position was held for two minutes. Next, the patient was seated and held in a sitting position for one minute. The resting sitting phase was designated as phase 1, the standing phase as phase 2, the standing phase as phase 3, and the seated phase as phase 4. Systolic blood pressure (SBP), mean blood pressure (MAP), and diastolic blood pressure (DBP) in each phase are shown in the figure. The patient did not meet the diagnostic criteria for orthostatic dysregulation (OD) or postural orthostatic tachycardia syndrome (POTS). No consistent trend could be observed in blood pressure fluctuations during each phase of treatment. SBP, MAP, and DBP, which were decreased before treatment, tended to increase with epipharyngeal abrasive therapy (EAT).

**Figure 5:**
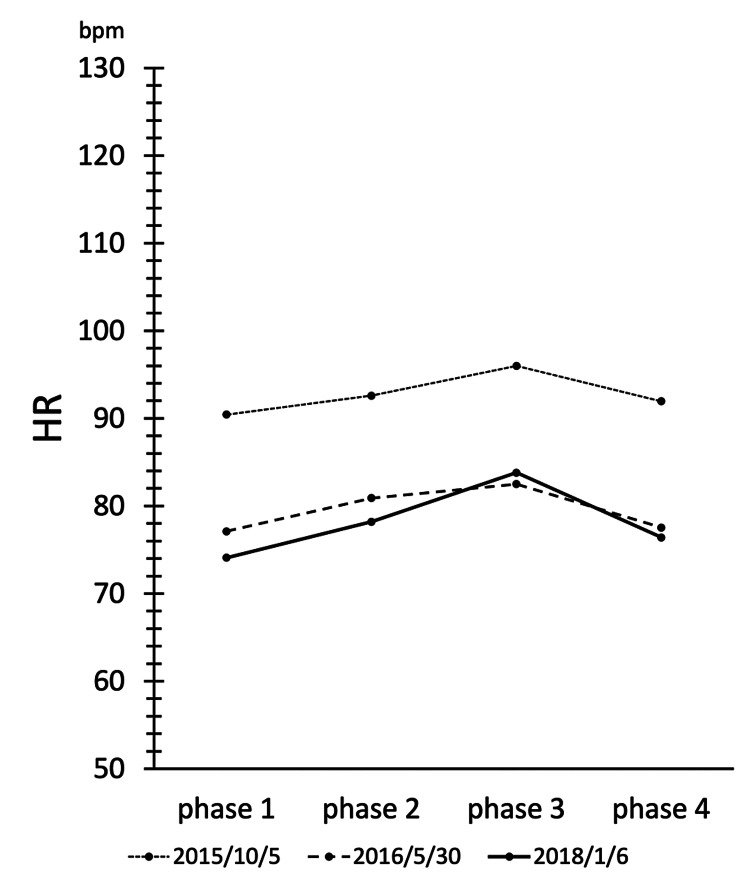
Changes in heart rate (HR) during the active standing test. In the patient's case, the HR, which was elevated before treatment, was shown to be reduced by epipharyngeal abrasive therapy (EAT).

Figure 4 shows the variation of SBP, MAP, and DBP measurements in each phase during the active orthostatic load test. EAT has been reported to have therapeutic effects on OD and POTS [7]. Although our patient did not meet the diagnostic criteria for OD or POTS, SBP, MAP, and DBP were shown to be affected by EAT. Although there was no consistent trend in the blood pressure fluctuations between phases, SBP, MAP, and DBP, which were decreased before treatment, tended to increase with EAT. It is thought that EAT activates the BR and stimulates sympathetic reflex activity, resulting in an increase in blood pressure during the orthostatic test. Figure 5 shows the variation of HR during each phase. In the present case, HR, which was elevated before treatment, was shown to be decreased by EAT. It has been reported that baseline HR is elevated in ME/CFS due to suppression of parasympathetic activity [12]. EAT may have the effect of decreasing HR by activating parasympathetic activity.

**Figure 6:**
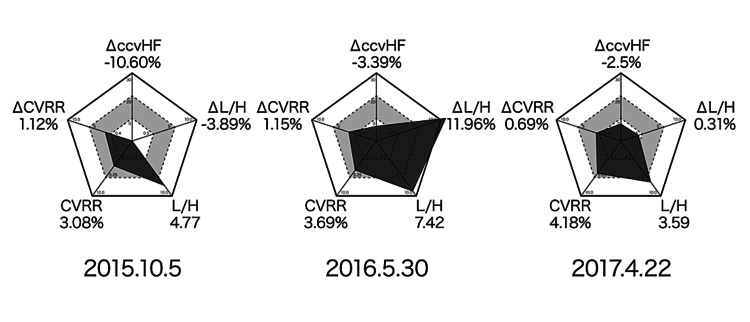
Changes in autonomic function over time. The diagram shows the change in autonomic function over time after epipharyngeal abrasive therapy (EAT). The gray area in the center of the diagram represents the mean ± 0.75 SD of the measurements of healthy adults of the same age group. The dark gray area indicates measurements of cases. ⊿ccv HF is an index of parasympathetic stress resilience, L/H is an index of sympathetic nervous system function tension, ⊿L/H is an index of sympathetic reflex stress detection (sympathetic switching), coefficient of variation on R-R interval (CVRR) is an index of total autonomic nervous system activity, and ⊿CVRR is an index of autonomic nervous system activity stress reactivity. A regular pentagon in the center of the diagram indicates an appropriate autonomic activity balance state; the balance of autonomic activity was adjusted by EAT enforcement.

Although HR is influenced by respiration and circulation, there are periodic fluctuations in the ECG R-R interval. Frequency analysis of these fluctuations divides them into a high-frequency (HF) component ranging from 0.15 to 0.40 Hz and a low-frequency (LF) component ranging from 0.04 to 0.15 Hz. HR variability analysis enables us to infer the parasympathetic and sympathetic components. The HF component mainly reflects the parasympathetic function, and the coefficient of component variance (ccv) HF is the coefficient of fluctuation of HF defined by Hayano et al. [13]. This ccv HF is used as an index of parasympathetic function, and the increase in ccv HF compared to the seated and resting state, or ⊿ccv HF, is used as an index of parasympathetic stress resilience. Since the LF component reflects sympathetic and parasympathetic functions, the LF/HF ratio (L/H), LF divided by HF, is used as an index of sympathetic nervous system function tension. The increase in L/H compared to sitting and standing, or ⊿L/H, is used as an index of the stress detection power of the sympathetic reflex (sympathetic switching power). Autonomic nerve activity (coefficient of variation on R-R interval [CVRR]) is an aggregate of HF, LF, and other components from frequency analysis results, and is used as an index of the sum of autonomic nerve activity. The increase in CVRR compared between sitting and standing, i.e., ⊿CVRR, is used as an index of stress reactivity of autonomic nerve activity. The five items of ⊿ccv HF, L/H, ⊿L/H, CVRR, and ⊿CVRR were used as evaluation items of autonomic function in this case [5]. Figure 6 shows the changes in autonomic function (Δccv HF, L/H, ΔL/H, CVRR, and ΔCVRR) over time after EAT. The gray area in the center of the diagram shows the mean ± 0.75 standard deviation (SD) of the measurements of healthy adults of the same age group. The dark gray area indicates the measured values of the cases. A regular pentagon in the center of the diagram indicates an appropriate balance of autonomic nervous system activity. The autonomic nervous system function was observed to recover parasympathetic activity and suppress excessive sympathetic reflexes, thereby regulating autonomic balance over time after the EAT was administered.

After returning to work, the patient has been followed up for approximately eight years to date and has had no relapse of CFS/ME. EAT findings after return to work are shown in Figure 1d. Endoscopic findings showed that the redness and swelling of the epipharyngeal mucosa had disappeared, and bleeding on rubbing was no longer observed. The patient was treated with EAT when symptoms of suspected flare-ups of chronic epipharyngitis, such as abnormal pharyngeal sensation in the epipharynx, appeared. The patient was treated for about six months before returning to work, and EAT was performed a total of 54 times, about twice a week. After returning to work, the patient continues to take EAT whenever she feels unwell. During the past seven years and six months, a total of 52 EATs have been performed.

## Discussion

Chronic epipharyngitis presents a variety of symptoms such as chronic fatigue in addition to symptoms such as posterior rhinorrhea and abnormal pharyngeal sensation. The usefulness of EAT as a treatment for chronic epipharyngitis has been reported [1]. EAT has also been reported to be effective for ME/CFS [1,7-8]. In the present report, EAT was performed in a case of chronic epipharyngitis with chronic fatigue as the main complaint, and the patient experienced improvement in chronic fatigue as well as in endocrine and autonomic function tests. There have been no reports of long-term follow-up of patients with chronic epipharyngitis with chronic fatigue or of changes in endocrine function tests and autonomic function tests as far as the author could find. Based on the experience of treating the present case, the mechanism of the effect of EAT on chronic fatigue was discussed from the perspective of endocrine and autonomic function, and the relationship between chronic epipharyngitis and ME/CFS was discussed.

Post-viral fatigue syndrome (PVFS) is a long-standing syndrome of unexplained general malaise, low-grade fever, headache, weakness, thinking disorder, and neuropsychiatric symptoms such as depression and sleep disturbance after viral infection [14]. PVFS presents with symptoms similar to those of ME/CFS. Studies of post-infection patients with mild or moderate COVID-19 infection have reported that about half of them meet the criteria for ME/CFS (long COVID-19). There is a global concern that PVFS will increase in the future [15]. Considering that ME/CFS occurs in clusters and is often associated with symptoms resembling infectious diseases, it is likely that some cases are triggered by infectious diseases. It is speculated that the weakening of immunity due to prior infections may be a factor [14].

According to the Japan Agency for Medical Research and Development (AMED) research group, the pathogenesis of ME/CFS is a condition based on the modulation of the nervous, endocrine, and immune systems related to environmental and genetic factors such as physical and mental stress. Various cytokine abnormalities induced by viral reactivation and chronic infections are thought to cause activation of microglia in the brain, and neuroinflammation in the brain induces brain dysfunction [6].

The Institute of Medicine (IOM) diagnostic criteria (2015) proposed systemic exertion intolerance disease (SEID). The following three symptoms are considered essential symptoms: (1) A sudden onset of severe general malaise of unknown cause, making it difficult for a person who has led a healthy life to continue a normal life; (2) feeling of intense fatigue after activity; and (3) inability to recover from fatigue through sleep. The presence of one or more of the following two symptoms constitutes the diagnostic criteria: (1) impairment of thinking and memory and (2) orthostatic intolerance [16].

The ME/CFS diagnostic criteria (2017) by the AMED study group combines symptom diagnosis and laboratory exclusion diagnosis and adopts (1) a minimum set of tests required for ME/CFS diagnosis, (2) description of major diseases and conditions to be differentiated, (3) description of diseases and conditions with coexisting conditions, and (4) assessment of quality of life (QOL) by performance status (PS). Symptoms persist for a certain period (usually six months or longer) and are evaluated to see if they have a significant impact on daily life [6]. Cases in which symptoms develop secondary to a confirmed infection are referred to as post-infection ME/CFS, but the treatment of cases in which the presence of chronic inflammation is suspected after the diagnosis of ME/CFS is made, as in this case, is unclear. There are also problems in distinguishing between diseases that should be excluded from ME/CFS and those that may coexist, as well as problems in differentiating ME/CFS from neuropsychiatric disorders. ME/CFS makes it difficult to detect abnormalities in common laboratory tests, and the problem is that no specific laboratory abnormalities have been shown to lead to a definitive diagnosis [17].

This is a case in which the diagnosis of ME/CFS was made at a ME/CFS clinic in Tokyo, but its presence was not initially apparent because the patient did not complain of typical symptoms of chronic epipharyngitis. The patient was referred to our clinic because of the lack of improvement in chronic fatigue. An endoscopic examination was performed at our hospital and a diagnosis of chronic epipharyngitis was made. Since chronic fatigue improved after treatment with EAT, this case was considered to have post-infection ME/CFS. However, since chronic fatigue is one of the symptoms of chronic epipharyngitis, it is possible that the underlying chronic epipharyngitis was the cause of chronic fatigue. Further investigation is needed to determine whether chronic epipharyngitis should be added to the list of diagnostic criteria for exclusion of ME/CFS, or whether chronic epipharyngitis is a disease that can coexist with ME/CFS.

At present, there is no effective curative treatment for all cases of ME/CFS, and adjustment of activity level (appropriate rest) and symptomatic treatment are currently being attempted. Medications and symptom management to reduce fatigue and pain, physical fitness and activity adjustment, stress management, psychotherapy, and counseling are being used. In the absence of an established treatment, it would be more beneficial to evaluate the effectiveness of treatment by EAT in the presence of chronic epipharyngitis.

In this case, endocrine function tests and autonomic function tests with HR variability analysis were performed to elucidate the mechanism of action of EAT and to evaluate the therapeutic effect of EAT on chronic fatigue. It has been reported that the HPA axis is abnormal in ME/CFS [18]. Based on the salivary cortisol test results in this case, EAT may have affected the HPA axis. Yamada reported that EAT promotes cortisol secretion and is effective in allergic rhinitis and collagen diseases [19]. The course of treatment in this case showed that EAT improved the morning surge of cortisol secretion and its diurnal variation. Normalization of cortisol secretion may lead to improvement of ME/CFS symptoms.

The epipharynx is rich in activated lymphocytes and autonomic nerve fibers. It is also a site prone to congestion, as indicated by the strong hemorrhage that occurs when the area is treated with abrasion. These findings suggest that the HPA axis is affected by mechanisms mediated by the immune system, the nervous system, and the cerebrospinal fluid venous circulatory system [20]. EAT may ameliorate chronic fatigue by improving dysfunction of the HPA axis.

Salivary amylase test results indicated that sympathetic reflex activity was increased in this patient. The endocrine autonomic reflexes in response to stress are the HPA system response and the SAM system response, and EAT affects both. Gender differences in the SAM system response have been observed, and it has been reported that the stimulatory effect is greater in women [4]. In the present case, the SAM system response was shown to be enhanced compared to healthy subjects. It is not possible to determine from the results of this case alone whether the SSA stimulation was a result of EAT-induced stimulation of the SAM system, or whether the SAM system response was enhanced by EAT because ME/CFS patients are predisposed to be more reactive to stress. However, EAT does affect endocrine reflexes.

The results of the orthostatic test in this case showed that EAT tended to increase blood pressure variability. EAT activates parasympathetic function, decreases HR, activates sympathetic function, and increases blood pressure during the orthostatic test. In other words, EAT may have the function of activating BR. The results of HR variability analysis showed that ⊿ccv HF tended to improve. The parasympathetic nervous system's stress resilience may have improved. L/H is an index of sympathetic nervous system function tension level, and a decreasing trend in tension level was observed. ⊿L/H, an index of stress detection power of sympathetic reflexes (sympathetic switching power), changed from no response to over-response and then to proper response. CVRR is a measure of the sum of autonomic activity, but autonomic activity was improved by EAT enforcement. ⊿CVRR is a measure of stress reactivity of autonomic activity but did not show a constant trend. In the early stage of treatment, parasympathetic activity was suppressed and imbalanced with sympathetic activity, but after continued EAT, parasympathetic reflexes recovered and sympathetic reflexes normalized with the improvement of excessive reactions. It is thought that the balance of autonomic nervous activity was adjusted by EAT, suggesting that EAT may have a balancing effect on autonomic nervous activity.

Parasympathetic and sympathetic responses are adaptive responses that maintain homeostasis in the body. In this case, the endocrine and autonomic reflexes were affected by EAT. EAT may have a therapeutic effect on chronic fatigue by influencing endocrine and autonomic reflexes. To apply EAT to the treatment of ME/CFS in the future, it is important to examine and diagnose, which regulatory mechanism of EAT is predominantly involved in each ME/CFS case and utilize it in the treatment of ME/CFS.

Concerning the pathogenesis of symptoms caused by chronic epipharyngitis: (1) symptoms due to local inflammatory stimuli such as pain, purulent discharge, and radiating pain caused by epipharyngitis; (2) symptoms associated with autoimmune and autoinflammatory diseases, such as IgA nephropathy, IgA vasculitis, arthritis, and palmoplantar pustulosis, which are caused by an immunological mechanism; (3) symptoms due to decreased cranial nerve function caused by impaired circulation in the brainstem, thalamus, and hypothalamus due to venous congestion of the epipharyngeal mucosa and cerebrospinal fluid congestion, accompanied by autonomic dysfunction, endocrine system dysfunction, and other disorders; (4) cases of autonomic overstimulation syndrome (Relly phenomenon) due to chronic autonomic nerve stimulation, stress reactions due to multiple vagal theory (Polyvagal theory), etc., may exist [7]. Chronic epipharyngitis is thought to have a variety of symptoms due to an additive combination of these pathogenic mechanisms. 67 Ga scintigraphy shows an accumulation in the nasopharynx even in healthy subjects, suggesting that the epipharynx is in a state of physiological inflammation. There may be cases of subclinical chronic epipharyngitis even if the onset of symptoms is not obvious [7].

The patient's habit of sniffing since childhood suggests that chronic epipharyngitis was the underlying cause. It is presumed that some mechanism was at work in this case to cause ME/CFS. The fact that the patient continued to visit the clinic after returning to society, complaining of poor physical condition when chronic epipharyngitis worsened, suggests that the presence of chronic epipharyngitis may have exacerbated ME/CFS. Since treatment of chronic epipharyngitis with EAT improved the symptoms of ME/CFS, it is possible that chronic epipharyngitis was involved as one of the causes of the onset of ME/CFS. This case suggests that EAT is effective for ME/CFS associated with chronic epipharyngitis.

ME/CFS has a few specific laboratory findings, and objective testing methods have not yet been established. In addition, many patients with EAT often drop out during treatment due to reasons such as severe pain, making it difficult to monitor the patient's progress over a long period. The shortcoming of this case report is that there is only one case. It is considered excessive to discuss the therapeutic efficacy of EAT for ME/CFS based on the efficacy of this case. It has been pointed out that microglial activation in the brain may induce brain inflammation in ME/CFS cases [6]. In the future, it is important to perform endocrine and autonomic function tests to verify the presence of neuroinflammation in the brain. This case is considered valuable as it involved the performance of EAT, endocrine function tests, and autonomic function tests, with the patient being followed up for an extended period. In the future, it will be necessary to increase the number of EAT cases and conduct further studies.

## Conclusions

This case report describes the experience of a patient with chronic epipharyngitis associated with chronic fatigue who underwent EAT and exhibited a positive treatment outcome. Various causes are considered for the development of ME/CFS, and chronic epipharyngitis is considered to be one of the causes of ME/CFS. At present, there is no effective treatment for all cases of ME/CFS, and objective testing methods have not yet been established. In this study, EAT was administered to a patient with ME/CFS, and improvement was observed in both subjective symptoms and objective findings of endocrine function tests and autonomic function tests. The mechanisms of action of EAT include local inflammation control, immunomodulation, and autonomic regulation. These three mechanisms of action may act synergistically to produce an effect on ME/CFS. Based on the experience of this case, it is thought that EAT may be one of the possible treatments for ME/CFS. In some cases of unexplained chronic fatigue, chronic epipharyngitis may potentially be present. When chronic epipharyngitis is suspected after endoscopic diagnosis, it is important to evaluate the patient with EAT, endocrine function tests, and autonomic function tests.
